# RP9 revisited; *RP9* p.(H137L) remains a likely cause of dominant splicing factor-Retinitis Pigmentosa

**DOI:** 10.1038/s41431-025-01964-0

**Published:** 2025-10-23

**Authors:** Leon Chang, James A. Poulter, Andrew R. Webster, Gavin Arno, Rajarshi Mukherjee, Andrew Lotery, Alison J. Hardcastle, Christopher M. Watson, Chris F. Inglehearn

**Affiliations:** 1 Leeds Institute of Medical Research, School of Medicine, University of Leeds, Leeds, UK; 2 NIHR Biomedical Research Centre, Moorfields Eye Hospital NHS Foundation Trust & UCL Institute of Ophthalmology, London, UK; 3 Department of Ophthalmology, St. James’s University Hospital, Leeds, UK; 4 Southampton Eye Unit, University Hospital Southampton, Southampton, UK; 5 Faculty of Medicine, University of Southampton, Southampton, UK; 6 North East and Yorkshire Genomic Laboratory Hub, Central Lab, St. James’s University Hospital, Leeds, UK

**Keywords:** Next-generation sequencing, Neurodegeneration

## Abstract

Variants in six pre-mRNA processing factors cause autosomal dominant Retinitis Pigmentosa (adRP). The *RP9* gene encodes a seventh splicing factor, and in 2002, we published *RP9* variants c.410A>T; p.(H137L) and c.509A>G; p.(D170G) as likely causes of adRP in a large multigenerational *RP9*-linked family and a single case, respectively. It has since been suggested these variants might be artefacts due to simultaneous amplification of the *RP9P* pseudogene, and no further pathogenic variants have been reported. We therefore rescreened two members of the *RP9*-linked family by genome sequencing. Examination of the 2 Mb locus defined by crossovers in the original family revealed no other plausible causative variants. Alignment of both short and long-read sequences confirmed that p.(H137L) is in the *RP9* gene, not the pseudogene. Screening for p.(H137L) in 1961 RP/Rod-cone dystrophy (RCD) cases from the Leeds patient cohort and UK 100,000 Genomes Project (100kGP) database revealed four further carriers. Including the original family, this variant was therefore present in 5/1962 RP/RCD probands, and is absent from gnomAD, constituting statistically significant enrichment in RP cases. Long-read sequencing of p.(H137L) in available carriers showed this is a UK founder allele. The *RP9* p.(D170G) allele was also confirmed as gene, not pseudogene, derived, but is present in 22 individuals in the 100kGP cohort, none with RP, as well as >200 individuals in gnomAD and Biobank, suggesting it is non-pathogenic. In conclusion, RP9 p.(H137L) is strongly associated with RP and remains the only plausible variant accounting for the condition in a large multi-generation adRP family.

## Introduction

In 2001, McKie et al. [1] reported that pathogenic variants in the gene encoding pre-mRNA processing factor PRPF8 (then named PRPC8) cause autosomal dominant retinitis pigmentosa (adRP). PRPF8 is a protein component of the spliceosome, a large, highly dynamic ribonucleoprotein complex comprised of five snRNPs and numerous proteins, which is thought to function as an enzyme, catalyzing the splicing of introns from heteronuclear RNA to produce mature mRNA molecules. This link between a splicing factor and a dominantly inherited Mendelian human condition affecting only the retina was unexpected, given the ubiquitous expression and essential housekeeping function of this highly conserved protein. Nevertheless, over the following decade, pathogenic variants causing adRP were identified in the genes encoding five further splicing factors, PRPF31 [2], PRPF3 [3], PRPF4 [4], PRPF6 [5], and SNRNP200 [6]. All of them, like PRPF8, are components of the U4/U6.U5 tri-snRNP subunit of the spliceosome. Recently, variants in the U4 and U6 snRNAs have also been reported as causing adRP [7]. However, it was not until 15 years after that initial report that a possible disease mechanism emerged, when a report linked these splicing-factor defects to the development of primary cilia, suggesting that splicing factor-related RP may be a form of ciliopathy [8].

**Fig. 1:**
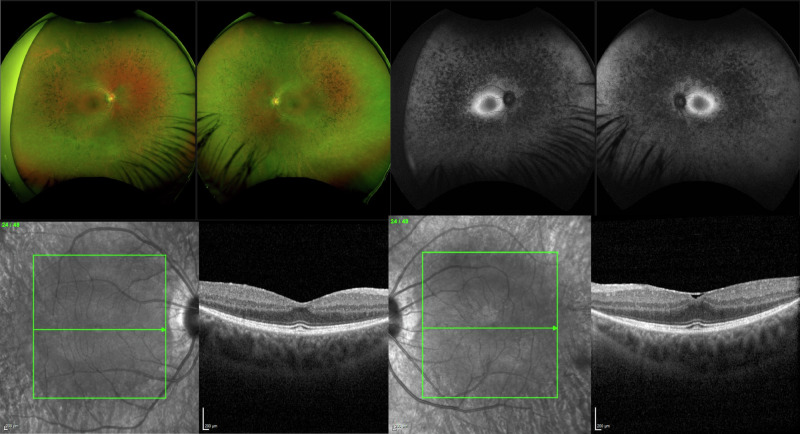
Clinical images from a member of the original RP9-linkedfamily. Ultrawide field pseudocolour fundus images, autofluorescence and OCT imaging in an affected individual in the mid-twenties, showing typical RP with retained retinal structure at the central macula, delineated by hyper-autofluorescent rings of Robson and a retained ellipsoid line (OCT). Outer-retinal degeneration has occurred anteriorly. RP was diagnosed in second decade, and the patient is night blind and unable to drive due to field loss, but has retained central vision. This is similar to RP seen in other forms of splicing factor RP.

In 1992, Jay et al. described a nine-generation extended UK family that included 46 living individuals with dominantly inherited RP [9]. RP in this family (Fig. 1) was described as showing variable expressivity, ranging from asymptomatic in late life to blindness in the third decade. Inglehearn et al. mapped the disease locus in this family to a region of chromosome 7p, which became known as the RP9 locus [10, 11], with a peak log of odds (LOD) score of 17.8 (>3 is considered significant). The same group subsequently identified a missense variant, c.410A>T; p.(H137L) (rs104894037), in a gene then known as *PAP-1*, now renamed *RP9*, as the likely cause of the disease, with a second *RP9* variant, c.509A>G; p.(D170G), identified in an unrelated sporadic RP case [12]. Later studies showed the encoded RP9 protein has a role in pre-mRNA splicing [13], is a component of the U4/U6.U5 tri-snRNP and interacts directly with PRPF3 [14]. The variable expressivity observed in the original *RP9*-linked family would also be consistent with it being a form of splicing factor-related RP, which is often characterized by variable penetrance, most notably in PRPF31-related disease [15]. The same phenomenon was reported recently in RP caused by variants in the U4 and U6 snRNAs [7]. Furthermore, two groups reported cellular models of RP9 disease. Jin et al. took induced pluripotent stem cells from patients carrying *RP9* c.410A>T; p.(H137L), differentiated them into rod-like cells and showed they overexpressed markers of oxidative and ER stress and were susceptible to degeneration in vitro [16]. More recently, Lv et al. used CRISPR/Cas9 gene editing to model the effects of both the equivalent missense variant and the knockout of *Rp9* in mouse 661-W cells, derived from mouse retina [17]. They observed decreased proliferation and migration in these cells, together with decreased expression and splicing of retinal genes. These studies suggest that RP due to variants in *RP9* may be another form of splicing factor-related adRP.

However, no cases of RP due to other variants in *RP9* have been reported since, in over 20 years of extensive research into the causes of inherited retinal diseases (https://web.sph.uth.edu/RetNet/). Furthermore, Sullivan et al. reported finding c.410A>T; p.(H137L) in an adRP case but concluded that it was an artefact resulting from PCR-amplification of the nearby, highly homologous *RP9P* pseudogene [18]. Significant doubt, therefore, remains as to the involvement of *RP9* variants in adRP.

Given the recent revolution in genetic technologies and new datasets that have become available since the publication of the study implicating *RP9* variants in adRP, we sought to re-investigate the *RP9* locus in the original linked family, sporadic case and other patients with RP, to determine whether *RP9* variants do indeed cause adRP or whether another variant, in this or another gene at the *RP9* locus, is the cause of retinal degeneration in this family.

## Methods

### Genome sequencing

Genome sequencing was performed on genomic DNA from blood, using the TruSeq Nano DNA HT Library Preparation Kit (Illumina, San Diego, CA, USA). Libraries were created according to manufacturer’s instructions. One sample was loaded per flow cell lane. Flow cells were loaded onto an Illumina HiSeq X sequencer and paired-end 150 bp sequencing was performed. Sequences were aligned to b37d5 human reference genome (human.g1k.v37) using the iSAAC aligner (v01.14.11.11) [19] to generate BAM files. Additional quality metrics were calculated using Picard WgsMetrics (v1.119). Structural and copy number variants were called in the linked region using MANTA (v1.2.0) and CANVAS (v1.28.0), respectively. Variant filtering was performed using vep_filter and remaining candidate variants were further checked for allele frequency using gnomAD (version 4.1) and gnomAD-SV.

### Exome sequencing

Exome sequencing was performed as part of the UK Inherited Retinal Disease Consortium. 200 ng genomic DNA was used to prepare libraries with the Agilent SureSelect AllExon kit using the v5 library. Pooled libraries were loaded onto an Illumina HiSeq3000 and paired-end 150 bp sequencing was performed. Data quality was assessed using FastQC (v0.11.6) and adaptors were trimmed using cutadapt (v1.12). Reads were aligned to bwa (v0.7.13) and processed according to GATK Best Practice guidelines for identifying germline mutations [20, 21]. Copy number analysis of exome data was performed using ExomeDepth [22], focussing on heterozygous variants in the RP9-linked region.

### Long-read nanopore sequencing

Primers for *RP9* p.H137L (Forward: AAGGGTCAAGGGACAGACCT, reverse: AAGCTCCCCAAGGAACAGAT; product 9253 bp) were designed for long-range PCR using the SequalPrep™ Long PCR Kit (ThermoFisher Scientific, Waltham, MA, USA). PCR was performed according to manufacturer’s instructions for 30 cycles using an annealing temperature of 56 °C and 0.5× Enhancer A. Amplification products were analysed on a 0.8% agarose gel and quantified by Qubit™ dsDNA BR Assay (ThermoFisher). Libraries were prepared using a ligation sequencing kit (SQK-LSK110), according to manufacturer’s instructions, for analysis on a Flongle flow cell (R9.4.1) (Oxford Nanopore Technologies, Oxford, UK). Up to 20 fmol each sample library was loaded onto individual Flongles and sequenced using a MinION. Raw FAST5 files were basecalled using Guppy (v5.2). Adaptor sequences were removed using Porechop (v0.2.4) and reads were aligned to the human reference genome using Minimap2 (v2.24). Sequencing metrics were collected using Nanostat (v1.4.0) and Samtools (v1.15) was used to split each bam file into 2000× read depth haplotypes for visualization using the IGV.

### Sanger sequencing

Two SNPs outside of and flanking the 2 Mb linked haplotype (GRCh38 SNP1 Chr7: g.32696283A>G; SNP2 Chr7: g.35162505C>A) were validated by Sanger sequencing. PCR was conducted using primers designed for SNP1 (forward: CTTCCCACGTCCATGTGTTC, reverse: ATAAAATCCTTGGTTCTCCCTTTTT; product size 248 bp) and SNP2 (forward: TGCTTTCCAGATTGCCACTG, reverse: GCTGTACCCTAAAGTCAAAACCA; product size 395 bp) and products were amplified using High-Fidelity Phusion Polymerase (LifeTech Scientific, Shenzhen, China), treated with ExoSAP-IT (ThermoFisher), then sequenced using the BigDye v3.1 terminator sequencing kit (Applied Biosystems).

### Association of variants in disease and control datasets

Data from the UK 100,000 Genomes Project (100kGP) were interrogated using the interactive variant analysis (IVA) tool within the research environment. Participants from the GRCh37 and GRCh38 rare disease germline studies were filtered for *RP9* p.(H137L) and p.(D170G) and queried for clinical features using HPO terms. Data were current as of December 2022 and were downloaded as part of GEL research project RR582, ‘Gene and Variant Discovery in Inherited Eye Diseases’. For interrogation of both variants in the UK Biobank dataset (490,640 individuals), the UK Biobank Allele Frequency Browser was used (https://afb.ukbiobank.ac.uk/), generated by the WGS consortium under the UK Biobank Resource (project ID 52293). Variant enrichment was assessed by Fisher’s exact test.

## Results

### Reanalysis of c.410A>T; p.(H137L)

To investigate whether variants elsewhere in the *RP9* locus could cause RP, we undertook genome sequencing in two affected individuals from the original linked family who are related via common ancestors seven generations ago and are therefore sixth cousins separated by fourteen meioses. Coverage and sequencing metrics for both individuals can be found in Supplementary Table 1. With the exception of RP9 p.(H137L), neither individual carried any variant with a minor allele frequency <0.001 and either a CADD (v1.7) score >15 or a SpliceAI score >0.4, in a gene implicated in adRP pathogenesis listed in the RetNet (https://web.sph.uth.edu/RetNet/) or Retigene [23] databases. Variants in other genes involved in adRP were therefore excluded. We then called all variants present within the linked locus (between STS makers D7S690-GS234F24CA1, (GRCh38) chr7:32,320,394-34,315,743 [12]) and filtered for heterozygous variants shared by both individuals with minor allele frequencies <0.001 in gnomAD, consistent with causing dominant disease. The remaining eleven SNVs and eight indels were annotated using variant effect predictor (NCBI). Manual interrogation of each variant found the eight indels were within non-coding microsatellite repeats and these were therefore excluded. The eleven SNVs were further scored using algorithms designed for both coding and non-coding variants (CADD v1.7 [24], FATHMM-mkl [25], ReMM [26]). All variants except *RP9* c.410A>T; p.(H137L) scored poorly (Table 1). Copy number variant (CNV) and structural variant (SV) analyses were performed using MANTA and CANVAS, but no potentially pathogenic CNVs or SVs were identified. The *RP9* variant was therefore the only plausible pathogenic variant identified within the RP9 locus.

**Table 1:** Bioinformatic prediction scores for all variants found in the heterozygous RP9-linked haplotype across the 2 mb locus shared by members of the original family and four apparently unrelated cases described here.

			FATHMM-MKL				ReMM	
Position (GRCh38)	Gene	CADD	Non-coding score	Non-coding groups	Coding score	Coding groups	Probability	Classification
chr7:32696283A>G	*DPY19L1P1*	7.7	0.15598	A	0.00457	AEFI	0.0845433	Low evidence
chr7:33096550T>A	*RP9*	24.8^a^	0.95405^a^	ABC	0.96568^a^	AEFBHCI	0.994029^a^	Deleterious^a^
chr7:33769044T>A		3.0	0.17276	A	0.09343	AEFI	0.567987	Low evidence
chr7:33969722A>G	*BMPER*	2.3	0.11395	AB	0.01480	AEFBI	0.656556	Low evidence
chr7:34559955A>C	*NPSR1-AS1*	1.8	0.06174	A	0.00105	AEFI	0.468994	Low evidence
chr7:34802348G>C	*NPSR1*	6.1	0.04398	A	0.00260	AEFI	0.0654325	Low evidence
chr7:34901635A>G		1.1	0.08234	AB	0.00702	AEFBI	0.0008333	Low evidence
chr7:34968054A>C	*DPY19L1*	4.1	0.12884	AD	0.03363	AEFDGI	0.002725	Low evidence
chr7:35062468G>A		3.2	0.06101	A	0.00333	AEFI	0.198626	Low evidence
chr7:35120873C>A	*DPY19L2P1*	2.3	0.04602	A	0.00170	AEFI	0.291211	Low evidence
chr7:35162505C>A	*DPY19L2P1*	0.4	0.07359	A	0.00501	AEFI	0.0	Low evidence

Full genotypes for all cases are shown in supplementary Table 2. Both coding and non-coding scores were used. FATHMM-MKL scores obtained from https://fathmm.biocompute.org.uk/fathmmMKL.htm are *p*-values in the range 0–1, with values above 0.5 predicted to be deleterious. Distinct feature groups (letters A-J) are used as predictors, with 10 groups of features, labeled A-J, used as predictive in coding regions, and a subset of 4 of these, A-D, also used in non-coding regions (detailed in ref. 25). ReMM (Regulatory Mendelian Mutation) scores were obtained from https://remm.bihealth.org/variant-lookup.

^a^Represents a score that is considered damaging/deleterious by the prediction tool.

**Fig. 2:**
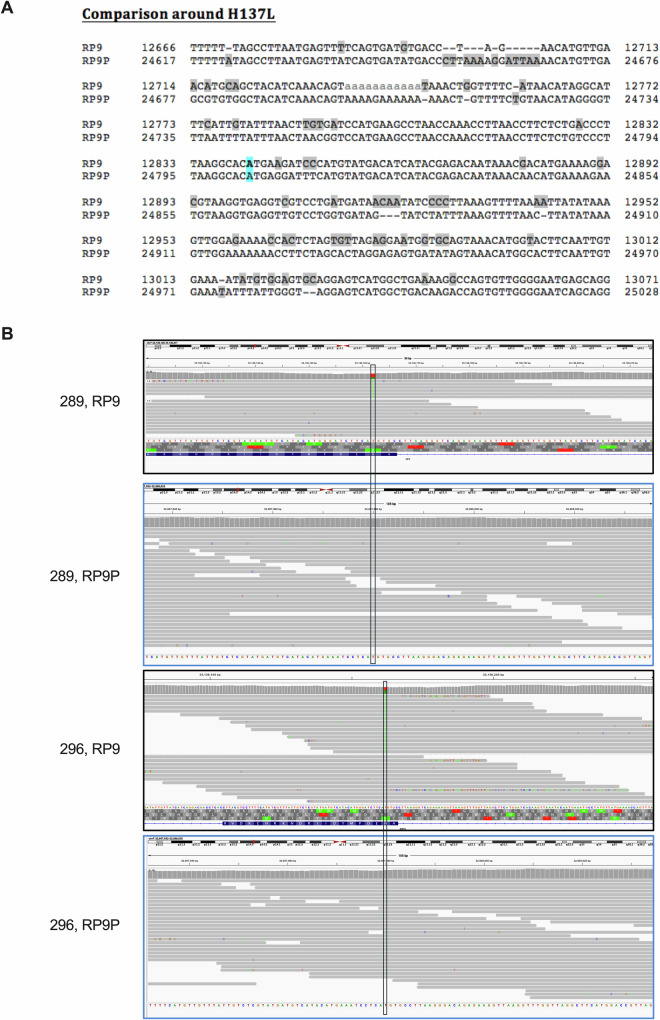
Comparative alignments between *RP9* and *RP9P* for a region centred upon the c.410A>T; p.(H137L) variant. **A** DNA sequence comparative alignment between *RP9* and *RP9P* for a region centred upon the c.410A>T; p.(H137L) variant, which is highlighted in cyan. Non-matching nucleotides are shaded. **B** IGV views of short read genome sequence from individuals RP9-289 and 296 at the *RP9* and *RP9P* regions, showing the T>A (forward strand, with the gene encoded on the reverse strand) change is present in *RP9* and not in *RP9P*.

In addition to *RP9*, a pseudogene (*RP9P*) is present within the disease-linked region defined by crossovers in the original linked family, 166 kb distal to *RP9*. The predicted *RP9P* mRNA shows 97% sequence identity over a 716 bp section encompassing the last four coding exons of the six-exon *RP9* transcript (NM_203288.2) and extending into the 3’ UTR. Both reported RP-linked variants are located within this interval. Furthermore, the 1 kb genomic region centred on exon 5 of *RP9* and extending into introns 4 and 5, containing the c.410A>T; p.(H137L) variant, shares 87.5% DNA sequence identity, as assessed by BLASTn (NCBI). We therefore aligned both the *RP9* and *RP9P* genomic DNA sequences and investigated the region surrounding the c.410A>T variant and 60 bp flanking either side (Fig. 2A). We found sufficient differences (11/121 bases) in the DNA sequence between *RP9* and *RP9P* to identify short-read sequences that were incorrectly mapped. Inspection in IGV of the reads covering c.410A>T in both affected individuals showed they mapped unambiguously to *RP9* and not *RP9P* (Fig. 2B). Given that the reference base in *RP9* is the same as the reference base in *RP9P*, we conclude that the c.410A>T variant is present in *RP9* and not *RP9P*.

**Fig. 3:**
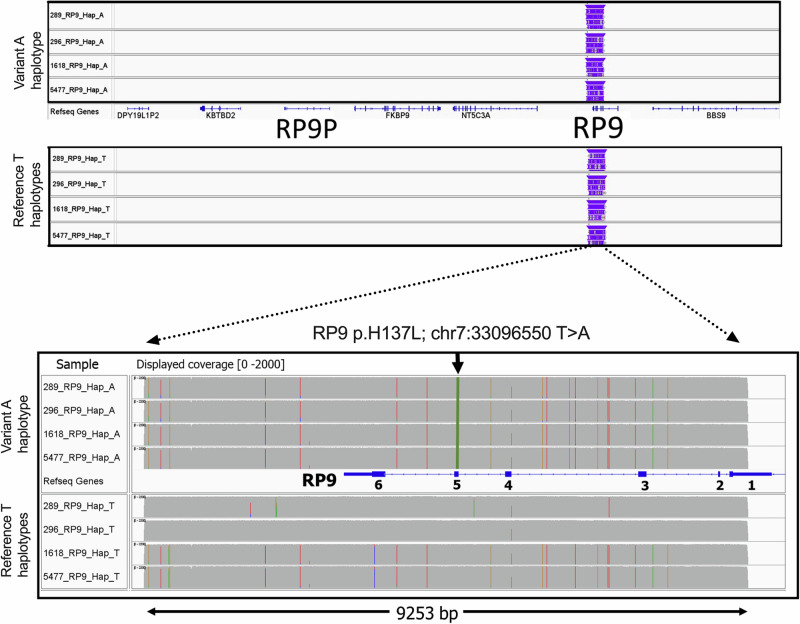
Long-read sequencing amplification products visualised using IGV. Haplotypes for four samples were created following the selection of reads that correspond to the T or A nucleotide at genomic position chr7:33096550. Variants defined by haplotype chr7: chr7:33096550 A are in linkage disequilibrium with the mutant allele. The upper two panels show the location of reads for each haplotype within a ~500 kb interval of the *RP9* locus, with a representation of the genes in the region shown between the two haplotype views; these data confirm that all reads map to *RP9* and not the pseudogene (*RP9P*). The lower panel magnifies the sequenced region; the upper of these show the conserved haplotype associated with the variant chr7:g.33096550A nucleotide, which is shared by all four cases analysed. The lower panel shows the varying haplotypes on the other, non-disease-associated allele. The genomic architecture of *RP9* is displayed between the two haplotype panels.

Ongoing genetic screening in 556 RP or rod-cone dystrophy (RCD) patients recruited either directly by the Leeds Vision Research Group or through collaborators identified two additional individuals with adRP carrying the c.410A>T; p.(H137L) variant in RP9 (laboratory numbers 1618 and 5477, Fig. 3; RP phenotype in 5477 shown in Supplementary Fig. 1). Two further cases were identified from the 1406 individuals recruited to the 100kGP with diagnoses of RP or RCD (Table 2). Including the original published family, this gives a total of five apparently unrelated cases/families with RP carrying this variant, out of 1962 (5/3924 alleles). By comparison, in gnomAD, *RP9* c.410A>T; p.(H137L) is absent from 831,084 alleles. This significant enrichment (*p* < 0.00001, Fisher’s exact test) in adRP/RCD cohorts provides further support for the pathogenicity of this variant.

**Table 2:** Recruited cases carrying RP9 p.H137L in 100KGP and their associated phenotype.

Cases of RP9 p.H137L in 100KGP							
Genome version	Genomic location	SNP	Gene	Individual (Sex)	Solved?	Disorders	Affection
GRCh38	7:33096550	rs104894037	RP9	1 (Male)	Yes	Ophthalmological disorder (Rod-Cone dystrophy)	Affected
				2 (Male)			Unaffected
GRCh37	7:33136162	rs104894037	RP9	3 (Male)	Yes	Ophthalmological disorder (Rod-Cone dystrophy)	Affected

The American College of Medical Genetics (ACMG) pathogenicity framework has introduced a standardized approach for the classification and interpretation of sequence variants [27]. Using ACMG criteria, the Franklin Genoox website (https://franklin.genoox.com/clinical-db/home) currently assesses *RP9* c.410A>T; p.(H137L) as a variant of unknown significance. PM2 (extremely low frequency in population) and PP3 (computational prediction tools unanimously support a deleterious effect) criteria are supportive but insufficient to classify the variant as pathogenic. However, based on the above enrichment, if PS4 (prevalence in affected individuals is significantly increased compared to the prevalence in controls) criteria are manually changed to ‘strongly supporting’, this assessment becomes likely pathogenic. If, in addition, PP1 (cosegregation with disease) criteria are manually changed to ‘very strong’ based on the presence of the p.(H137L) variant in all 46 affected members of the original RP9-linked family, this assessment becomes pathogenic. However, it should be noted that this criterion will apply to any variant within the co-segregating haplotype. Altogether, these data support the hypothesis that the c.410A>T; p.(H137L) variant is the cause of RP in the original RP9-linked family and additional cases.

We speculated that the new cases identified in the Leeds and 100kGP cohorts had not arisen independently but rather were relatives of the same extended pedigree. To test this hypothesis, a 9253 bp amplimer centred on the *RP9* c.410A>T variant was amplified in the same two members of the original RP9-linked family and in the two additional carriers identified in the Leeds cohort, and sequenced by ONT nanopore sequencing. This amplimer contained the entire coding sequence of RP9 transcript NM_203288.2 and extended over 3 kb downstream. Long-read sequencing once again confirmed that this variant is present in *RP9* and not the *RP9P* pseudogene (Fig. 3). Furthermore, analysis of the 9253 bp amplimer allowed the sorting of variant from non-variant alleles and revealed a complex haplotype of local SNPs shared by all chromosomes carrying the c.410A>T variant, distinct from the second (non-variant carrying) chromosomes in each case (Fig. 3). To confirm that this haplotype covered the entire RP9 locus in cases 1618 and 5477, identified in the Leeds IRD cohort, flanking SNPs 1 and 2 (Chr7:g.32696283A>G and Chr7:g.35162505C>A) were genotyped, and the RP9 associated allele was confirmed in each case (Supplementary Fig. 2). It was not possible to obtain DNA for the two additional cases identified in the 100kGP cohort, and the short-read genome sequence carried out by Genomes England does not allow accurate haplotyping. However, the SNP genotypes observed in these cases (Supplementary Table 2) are consistent with these individuals also carrying the c.410A>T variant on the same local haplotype. It therefore seems likely that this is a founder allele that arose in the UK population.

### Reanalysis of p.D170G

A second variant in *RP9*, c.509A>G; p.(D170G), was identified in a single RP case in the original report [12]. To determine whether this remained the most likely cause of RP in this individual, we performed exome sequencing and filtered for rare variants in known IRD genes. Due to lack of family history of RP for this female patient, we looked for variants that fit autosomal dominant or autosomal recessive modes of inheritance. None that could account for disease was identified except the *RP9* variant.

To assess the extent of homology between *RP9* and *RP9P* in the region surrounding this variant, we again aligned the variant and flanking 60 bp of sequence either side (Supplementary Fig. 3). For this variant, the adjacent DNA sequence is identical in the *RP9* gene and *RP9P* pseudogene except for the variant site itself. Analysis of the c.590A>G variant in the pseudogene revealed that the alternative allele (G) for this variant is the reference base for *RP9P*. It was therefore plausible that both alleles of *RP9* were wild-type and the appearance of a variant may have arisen as a sequencing artefact due to co-amplification of the homologous region of *RP9P*. To test this hypothesis, we designed primers that were specific to *RP9* and to *RP9P* (Supplementary Fig. 2), amplified both regions by PCR and performed Sanger sequencing. This confirmed that the c.509A>G variant was indeed present and heterozygous in *RP9* in this individual.

The p.(D170G) variant has previously been shown to affect phosphorylation of RP9 and, in turn, splicing efficiency, which supports the potential pathogenicity of this variant [13]. However, 26 carriers were identified in the 100kGP cohort, none with a retinal disease (Supplementary Table 3). Furthermore, in the latest update of gnomAD, this variant is observed in 101 of 831,084 alleles for which sequence is available, while in the UK Biobank cohort, it is present in 136 of 981070 alleles. While this implies a population frequency of 0.00013 (1 in 7692 alleles), the prevalence of all forms of RP is estimated to be only 1/4000 [28]. These observations, therefore, suggest it is unlikely this variant is the cause of dominant RP.

**Fig. 4:**
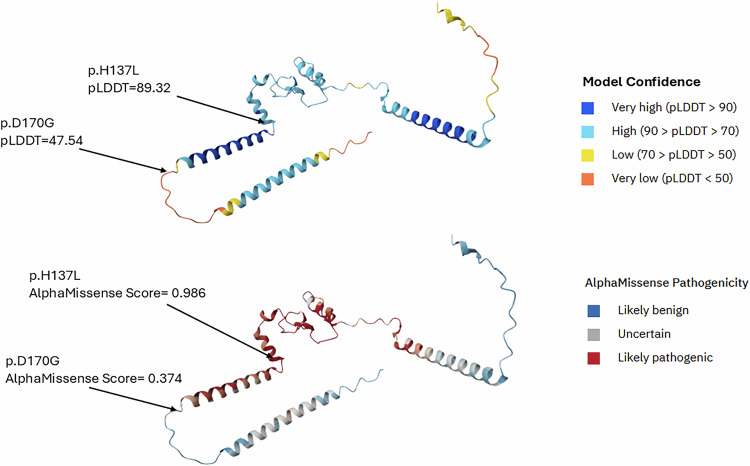
AlphaFold (upper panel) and AlphaMissense (lower panel) analysis of the RP9 protein. The sites of the c.410A>T; p.(H137L) and c.509A>G; p.(D170G) variants are denoted by arrows.

Analysis of the p.(D170G) and p.(H137L) variants in the AlphaFold protein structure database [29] (Fig. 4) further supports these conclusions. While the confidence score for the complete structure is low (pTM = 0.25), the predicted local secondary structures have higher confidence scores. The pLDDT score, a per-residue measure of local confidence, is 89.32 for p.(H137L), but only 47.54 for p.(D170G), suggesting the former lies in a constrained protein domain while the latter is in a more disordered region. The accompanying AlphaMissense pathogenicity scores (range 0–1), which integrate structural context with evolutionary conservation, predict p.(H137L) is likely pathogenic (0.986), but p.(D170G) is of uncertain pathogenicity (0.374).

## Discussion

Over the last 30 years, there has been a huge increase in understanding of how genetic variants cause or contribute to human diseases, driven in large part by the human genome project [30], the development of massively parallel sequencing [31] and large-scale genome sequencing programmes such as the 100kGP [32]. This has created an extensive literature documenting variants in specific genes that cause a Mendelian inherited disease, as well as increasing our understanding of the underlying biology. Most causal links between gene, variant and disease are well established, with multiple documented cases and additional evidence from functional cell and molecular biology experiments. However, this literature also contains instances of proposed causative links that remain unsubstantiated by further genetic or molecular data. This can confound the interpretation of genomic data by diagnosticians, preventing identification of the variants involved or even leading to incorrect genetic diagnoses.

The association of *RP9* variants with dominant Retinitis pigmentosa is one such link. Limited genetic data, first reported 23 years ago [12], suggested but did not prove causation. Subsequently, the encoded protein was shown to be a component of the spliceosome [14], other variants of which cause adRP [2–7, 33]. Furthermore, the *RP9-*associated phenotype shows incomplete penetrance, consistent with other forms of splicing factor-related RP, and molecular and cellular assays suggested a splicing defect with potentially pathogenic consequences at the cellular level [16, 17]. However, no further variants have been identified, and some functional assays yielded contradictory findings, as noted in OMIM. Furthermore, the presence of a nearby *RP9P* pseudogene led to the suggestion that the reported *RP9* variants may be artefacts caused by co-amplification of gene and pseudogene, or misalignment of reads between these highly homologous sequences [18]. The published genetic analysis of the *RP9* locus was informed only by the earliest draft of the human genome, and was carried out before the development of massively parallel sequencing. In light of these concerns, we concluded there was a need to revisit existing data on *RP9* variants as a cause of adRP using contemporary sequencing and bioinformatics approaches.

In this study, we show that *RP9* c.410A>T; p.(H137L) remains the most likely cause of adRP in the original RP9-linked family and report four further UK probands with RP also found to carry it. We show that it is the only plausible pathogenic variant identified following genomic sequencing of the locus in the RP9-linked family; it is located in the gene and not the pseudogene; it is significantly enriched in patients with RP over population controls; it is a founder variant in the UK; and, in light of these findings, ACMG criteria support classification of this variant as likely pathogenic. However, we also show that RP9 c.509A>G; p.(D170G), the second variant reported in the original paper [12], is present in 22 people recruited to the 100kGP, none of whom were annotated as having an ocular phenotype via HPO terms submitted at the time they were recruited, and in over 200 individuals in the latest gnomAD and UK Biobank data releases. ACMG criteria, therefore, classify this variant as a VUS, and its role in causing adRP must be considered substantially in doubt. A similar situation exists with regard to Doyne macular dystrophy, where a single founder variant, c.1033 C > T; p.R345W in EFEMP1, causes the condition, with no further variants in this gene having been implicated [34].

Given the ACMG reclassification, we suggest these data are now sufficiently compelling to counsel RP patients carrying the UK founder c.410A>T; p.(H137L) variant in *RP9* to the effect that this is the likely cause of their condition. By contrast, the RP9 c.509A>G; p.(D170G) variant remains a VUS. This has significant clinical implications for patients, relatives and the clinicians and diagnosticians working with them, clarifying recurrence risk, excluding other potential causes and allowing clearer prognoses based on the previously published phenotype observed in this family (Fig. 1) [9, 35] (family 2).

Further variants in *RP9* that segregate with adRP and/or are confirmed by functional assays as potentially pathogenic would provide additional support for causation. The gene is included in the Genomics England PanelApp Retinal Disorders panel and in other laboratory screening panels around the world, and ClinVar records a nonsense and a frameshift variant in *RP9* as likely pathogenic based on their having been identified in RP patients in this way. c.484_485del, (p.Gln162fs) is in sequence which is near identical in *RP9* and *RP9P*, but is not the reference sequence in either, meaning it could be occurring in the pseudogene rather than the gene. It occurs 22 times in gnomAD, making it less plausible as a cause for a partial penetrance dominant disease. c.5C>A, (p.Ser2Ter) is in sequence uniquely found in the RP9 gene and not the pseudogene, and occurs only once in gnomAD, and is therefore a plausible RP-causing variant. However, these findings have not been peer-reviewed and no further information is supplied, so these results remain tentative. Furthermore, gnomAD records many other *RP9* missense variants, as well as a number of rare frameshift and nonsense variants, a stop-loss with an allele frequency of ~0.01 and a chr7:33090225-33147816 duplication encompassing the entire gene, with an allele frequency of ~0.002. As a consequence, the gnomAD constraint metrics for this gene suggest variants are tolerated, implying that RP is not caused by RP9 haploinsufficiency or gene dosage. Instead, this could suggest a specific role for the affected residue, or a specific consequence if it is changed, pointing to a possible gain-of-function disease mechanism.

We suggest there is a need for other researchers with access to DNA from historical studies that do not meet current pathogenicity criteria to undertake similar analyses, in order to refine the published understanding of human inherited diseases, and in some cases to remove erroneous or unproven associations between variant and disease. This effort will reduce ambiguity in diagnostic testing and allow more rapid and efficient identification of true pathogenic variants in human DNA sequence.

## Supplementary information

Supplementary materials.

## Data Availability

Data available on request from the author. The *RP9* c.410A>T; p.(H137L) variant has been submitted to ClinVar with a new classification linked to this paper (ClinVar Submission ID: SUB15667213, accession number pending).

## References

[1] McKie AB, McHale JC, Keen TJ, Tarttelin EE, Goliath R, van Lith-Verhoeven JJ, et al. Mutations in the pre-mRNA splicing factor gene PRPC8 in autosomal dominant retinitis pigmentosa (RP13). Hum Mol Genet. 2001;10:1555–62. 11468273 10.1093/hmg/10.15.1555

[2] Vithana EN, Abu-Safieh L, Allen MJ, Carey A, Papaioannou M, Chakarova C, et al. A human homolog of yeast pre-mRNA splicing gene, PRP31, underlies autosomal dominant retinitis pigmentosa on chromosome 19q13.4 (RP11). Mol Cell. 2001;8:375–81. 11545739 10.1016/s1097-2765(01)00305-7

[3] Chakarova CF, Hims MM, Bolz H, Abu-Safieh L, Patel RJ, Papaioannou MG, et al. Mutations in HPRP3, a third member of pre-mRNA splicing factor genes, implicated in autosomal dominant retinitis pigmentosa. Hum Mol Genet. 2002;11:87–92. 11773002 10.1093/hmg/11.1.87

[4] Chen X, Liu Y, Sheng X, Tam PO, Zhao K, Chen X, et al. PRPF4 mutations cause autosomal dominant retinitis pigmentosa. Hum Mol Genet. 2014;23:2926–39. 24419317 10.1093/hmg/ddu005

[5] Tanackovic G, Ransijn A, Ayuso C, Harper S, Berson EL, Rivolta C. A missense mutation in PRPF6 causes impairment of pre-mRNA splicing and autosomal-dominant retinitis pigmentosa. Am J Hum Genet. 2011;88:643–9. 21549338 10.1016/j.ajhg.2011.04.008 PMC3146730

[6] Zhao C, Bellur DL, Lu S, Zhao F, Grassi MA, Bowne SJ, et al. Autosomal-dominant retinitis pigmentosa caused by a mutation in SNRNP200, a gene required for unwinding of U4/U6 snRNAs. Am J Hum Genet. 2009;85:617–27. 19878916 10.1016/j.ajhg.2009.09.020 PMC2775825

[7] Quindoz M, Rodenburg K, Cvackova Z, Kaminska K, De Bruijn SE, Iglasias-Romero AB, et al. *De novo* and inherited dominant variants in U4 and U6 snRNAs cause retinitis pigmentosa. Preprint at. 2025. 10.1101/2025.01.06.24317169. 10.1038/s41588-025-02451-4 PMC12807869 41513982

[8] Wheway G, Schmidts M, Mans DA, Szymanska K, Nguyen TT, Racher H, et al. An siRNA-based functional genomics screen for the identification of regulators of ciliogenesis and ciliopathy genes. Nat Cell Biol. 2015;17:1074–87. 26167768 10.1038/ncb3201 PMC4536769

[9] Jay M, Bird AC, Moore AN, Jay B. Nine generations of a family with autosomal dominant retinitis pigmentosa and evidence of variable expressivity from census records. J Med Genet. 1992;29:906–10. 1479605 10.1136/jmg.29.12.906 PMC1016211

[10] Inglehearn CF, Carter SA, Keen TJ, Lindsey J, Stephenson AM, Bashir R, et al. A new locus for autosomal dominant retinitis pigmentosa on chromosome 7p. Nat Genet. 1993;4:51–53. 8513323 10.1038/ng0593-51

[11] Inglehearn CF, Keen TJ, Al-Maghtheh M, Gregory CY, Jay MR, Moore AT, et al. Further refinement of the location for autosomal dominant retinitis pigmentosa on chromosome 7p (RP9). Am J Hum Genet. 1994;54:675–80. 8128965 PMC1918098

[12] Keen TJ, Hims MM, McKie AB, Moore AT, Doran RM, Mackey DA, et al. Mutations in a protein target of the Pim-1 kinase associated with the RP9 form of autosomal dominant retinitis pigmentosa. Eur J Hum Genet. 2002;10:245–9. 12032732 10.1038/sj.ejhg.5200797

[13] Maita H, Kitaura H, Keen TJ, Inglehearn CF, Ariga H, Iguchi-Ariga SM. PAP-1, the mutated gene underlying the RP9 form of dominant retinitis pigmentosa, is a splicing factor. Exp Cell Res. 2004;300:283–96. 15474994 10.1016/j.yexcr.2004.07.029

[14] Maita H, Kitaura H, Ariga H, Iguchi-Ariga SM. Association of PAP-1 and Prp3p, the products of causative genes of dominant retinitis pigmentosa, in the tri-snRNP complex. Exp Cell Res. 2005;302:61–68. 15541726 10.1016/j.yexcr.2004.08.022

[15] Al-Maghtheh M, Vithana E, Tarttelin E, Jay M, Evans K, Moore T, et al. Evidence for a major retinitis pigmentosa locus on 19q13.4 (RP11) and association with a unique bimodal expressivity phenotype. Am J Hum Genet. 1996;59:864–71. 8808602 PMC1914817

[16] Jin ZB, Okamoto S, Osakada F, Homma K, Assawachananont J, Hirami Y, et al. Modelling retinal degeneration using patient-specific induced pluripotent stem cells. PLoS ONE. 2011;6:e17084. 21347327 10.1371/journal.pone.0017084 PMC3037398

[17] Lv JN, Zhou GH, Chen X, Chen H, Wu KC, Xiang L, et al. Targeted RP9 ablation and mutagenesis in mouse photoreceptor cells by CRISPR-Cas9. Sci Rep. 2017;7:43062. 28216641 10.1038/srep43062 PMC5317003

[18] Sullivan LS, Bowne SJ, Birch DG, Hughbanks-Wheaton D, Heckenlively JR, Lewis RA, et al. Prevalence of disease-causing mutations in families with autosomal dominant retinitis pigmentosa: a screen of known genes in 200 families. Invest Ophthalmol Vis Sci. 2006;47:3052–64. 16799052 10.1167/iovs.05-1443 PMC2585061

[19] Raczy C, Petrovski R, Saunders CT, Chorny I, Kruglyak S, Margulies EH, et al. Isaac: ultra-fast whole-genome secondary analysis on Illumina sequencing platforms. Bioinformatics. 2013;29:2041–3. 23736529 10.1093/bioinformatics/btt314

[20] McKenna A, Hanna M, Banks E, Sivachenko A, Cibulskis K, Kernytsky A, et al. The genome analysis toolkit: a mapreduce framework for analyzing next-generation DNA sequencing data. Genome Res. 2010;20:1297–303. 20644199 10.1101/gr.107524.110 PMC2928508

[21] DePristo MA, Banks E, Poplin R, Garimella KV, Maguire JR, Hartl C, et al. A framework for variation discovery and genotyping using next-generation DNA sequencing data. Nat Genet. 2011;43:491–8. 21478889 10.1038/ng.806 PMC3083463

[22] Plagnol V, Curtic J, Epstein M, Mok KY, Stebbings E, Grigoriadou S, et al. A robust model for read count data in exome sequencing experiments and implications for copy number variant calling. Bioinformatics. 2012;28:2747–54. 22942019 10.1093/bioinformatics/bts526 PMC3476336

[23] Rivolta C, Celik E, Kamdar D, Cancellieri F, Kaminska K, Ullah M, et al. RetiGene, a comprehensive gene atlas for inherited retinal diseases. Am J Hum Genet. 2025;112:2253–65. 10.1016/j.ajhg.2025.08.017 PMC12696501 40961941

[24] Kircher M, Witten DM, Jain P, O’Roak BJ, Cooper GM, Shendure J. A general framework for estimating the relative pathogenicity of human genetic variants. Nat Genet. 2014;46:310–5. 24487276 10.1038/ng.2892 PMC3992975

[25] Shihab HA, Gough J, Cooper DN, Stenson PD, Barker GL, Edwards KJ, et al. Predicting the functional, molecular, and phenotypic consequences of amino acid substitutions using hidden Markov models. Hum Mutat. 2013;34:57–65. 23033316 10.1002/humu.22225 PMC3558800

[26] Schubach M, Nazaretyan L, Kircher M. The regulatory Mendelian mutation score for GRCh38. Gigascience. 2022;12:giad024. 37083939 10.1093/gigascience/giad024 PMC10120424

[27] Richards S, Aziz N, Bale S, Bick D, Das S, Gastier-Foster J, et al. Standards and guidelines for the interpretation of sequence variants: a joint consensus recommendation of the American College of Medical Genetics and Genomics and the Association for Molecular Pathology. Genet Med. 2015;17:405–24. 25741868 10.1038/gim.2015.30 PMC4544753

[28] Hamel C. Retinitis pigmentosa. Orphanet J Rare Dis. 2006;1:40. 17032466 10.1186/1750-1172-1-40 PMC1621055

[29] Cheng J, Novati G, Pan J, Bycroft C, Žemgulytė A, Applebaum T, et al. Accurate proteome-wide missense variant effect prediction with AlphaMissense. Science 2023;381:eadg7492. 10.1126/science.adg7492 37733863

[30] Lander ES, Linton LM, Birren B, Nusbaum C, Zody MC, Baldwin J, et al. Initial sequencing and analysis of the human genome. Nature. 2001;409:860–921. 10.1038/35057062 11237011

[31] Shendure J, Ji H. Next-generation DNA sequencing. Nat Biotechnol. 2008;26:1135–45. 18846087 10.1038/nbt1486

[32] The National Genomic Research Library v5.1, Genomics England. 10.6084/m9.figshare.4530893.v7.

[33] Mordes D, Luo X, Kar A, Kuo D, Xu L, Fushimi F, et al. Pre-mRNA splicing and retinitis pigmentosa. Mol Vis. 2006;26:1259–71. PMC2683577 17110909

[34] Stone EM, Lotery AJ, Munier FL, Heon E, Piguet B, Guymer RH, et al. A single EFEMP1 mutation associated with both malattia leventinese and Doyne honeycomb retinal dystrophy. Nat Genet. 1999;22:199–202. 10369267 10.1038/9722

[35] Moore AT, Fitzke F, Jay M, Arden GB, Inglehearn CF, Keen TJ, et al. Autosomal dominant retinitis pigmentosa with apparent incomplete penetrance: a clinical, electrophysiological, psychophysical, and molecular genetic study. Br J Ophthalmol. 1993;77:473–9. 8025041 10.1136/bjo.77.8.473 PMC504578

