# Supplementary material for: RP9 revisited; RP9 p.(H137L) remains a likely cause of dominant splicing factor-Retinitis Pigmentosa
Source: Eur J Hum Genet. 2025 Oct 23;34(2):227–35. doi: 10.1038/s41431-025-01964-0 (PMC12858846; doi:10.1038/s41431-025-01964-0)
Supplement: Supplementary file 1 — Supplementary materials. [file 41431_2025_1964_MOESM1_ESM.docx]

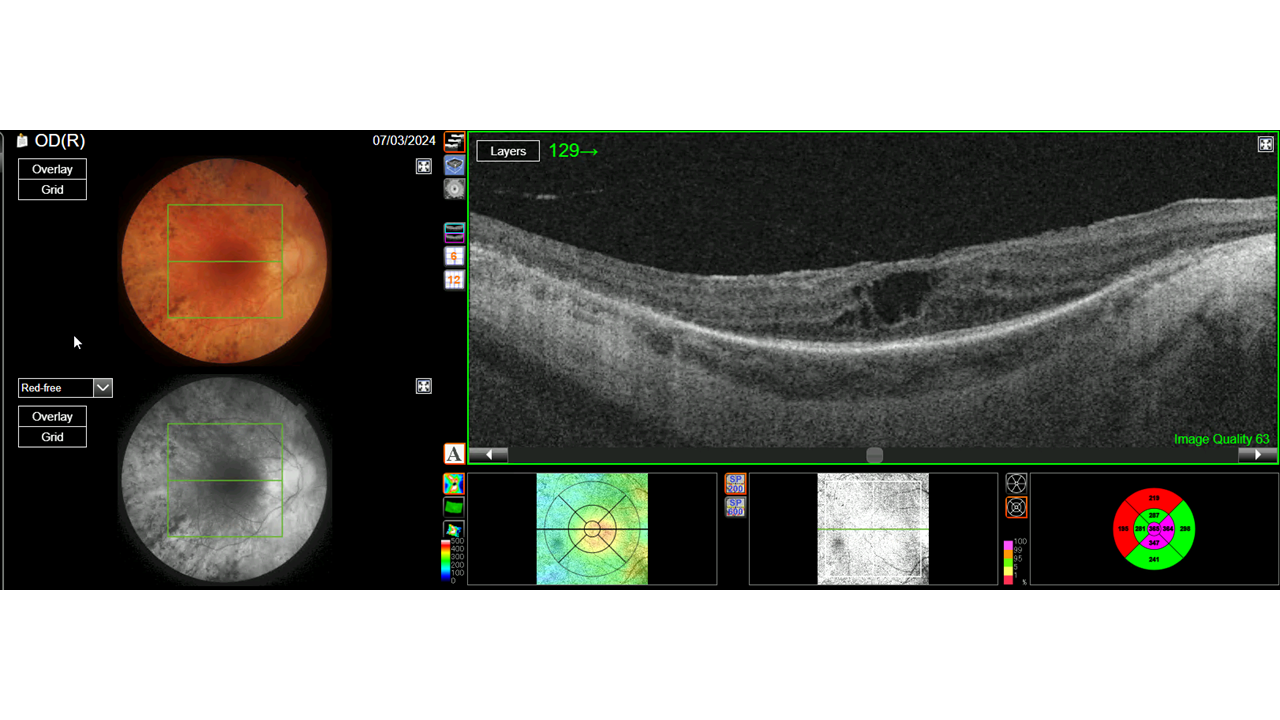

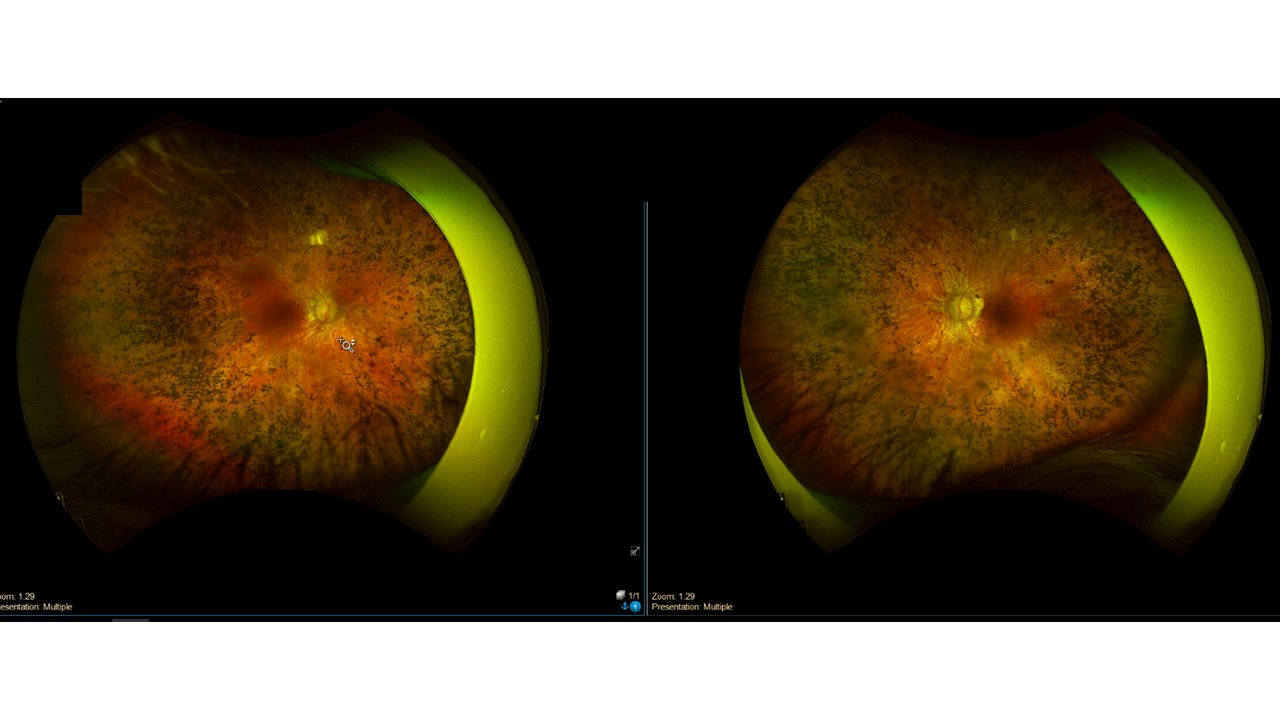


**Supplementary Figure 1.** Ultrawide field pseudocolour fundus images, autofluorescence and OCT imaging from case 5477, obtained in the 6th decade of life.


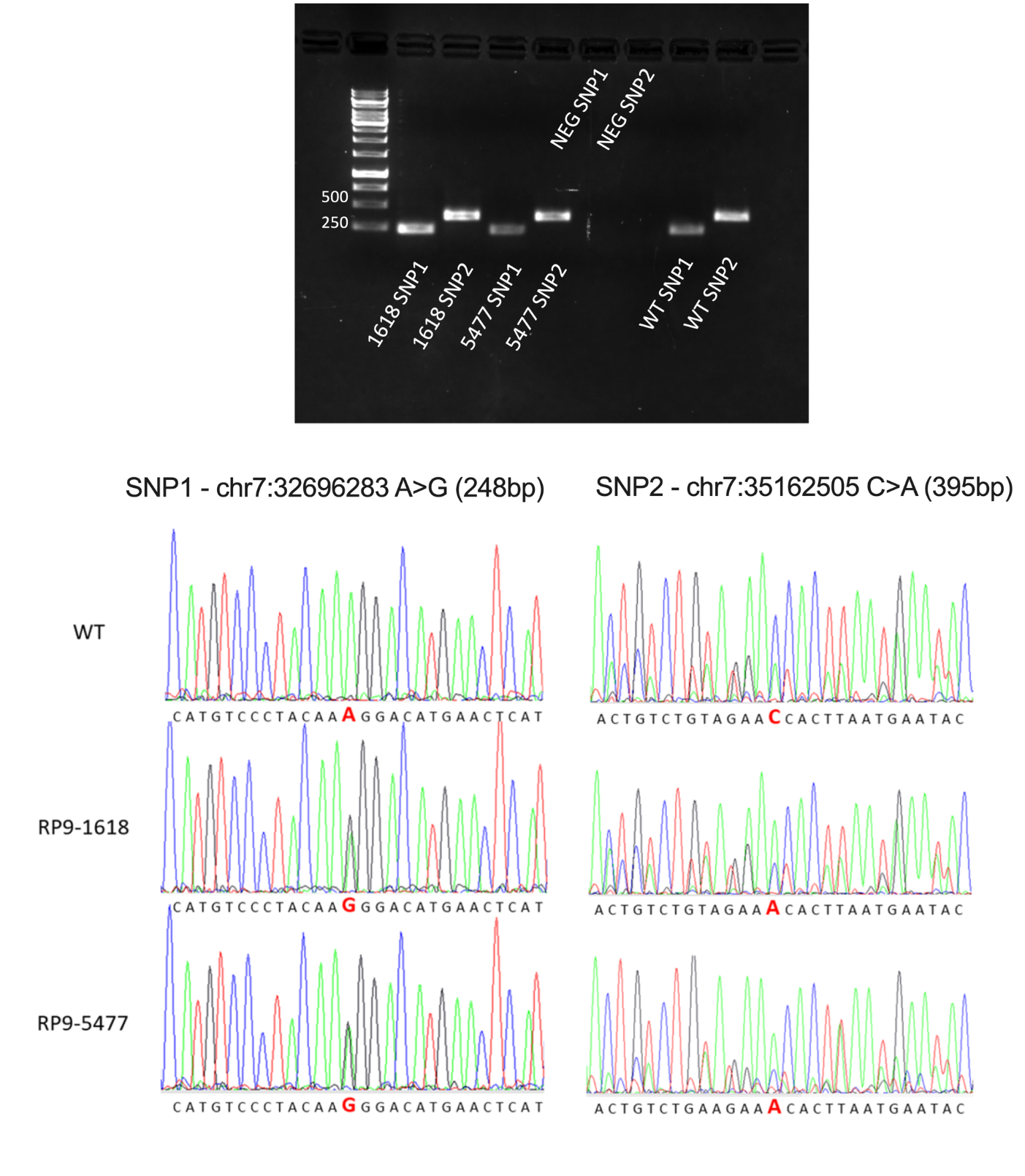


**Supplementary Figure 2.** Upper image shows PCR amplification of the SNPs flanking the RP9 locus in the two individuals identified via cohort screening for variant c.410A>T; p.(H137L), and in a wild-type control. Lower images show electropherogram traces from these individuals, which confirms that both variant c.410A>T; p.(H137L) carriers, unlike the WT control, are heterozygous for the rare non-reference allele at these SNPs. These individuals were analysed by long read sequencing (Figure 3), but were not subject to genome sequencing, so flanking SNPs were used to confirm the presence of the extended RP9 haplotype across the entire locus.

**Comparison between RP9 and RP9P around D170G (shaded bases show differences)**

RP9 gacagaggaagaccctgtctctaaaaaaaaaagaaaaaaatt**agacatgattagagagag**

RP9P ----------------------------taaagattatacctagacaagattagaga---

RP9 **gtgga**aaaaattggagggtttgttttttcaaattggtggca------------ttataac

RP9P -gtgaataatcaggagagtttgttttttcaaactggttacattaaattataattgttgag

RP9 tgttaaagatgtagattaagaaattgagcagtaggggaatggtttagtaaagtatgcgtc

RP9P ggtgtagattatctattaggaaattgagcagttggggaatggtttagtaaagtatgcgtc

RP9 tgttagaaattacaattgtaaggactgtggaaacatgcagaaatgtctaggacaataaaa

RP9P tgttagaatttataattgtaagtactgtgggaacctacagaaatgactaggacaaggaaa

RP9 agaatacaaagt-ggcttgtacactggtttcagtcacgtaaagta-ggcattcaggtgga

RP9P agaatacaaaaatggtttgtacactggttacagtcacataaacataggcattcaggtgaa

RP9 gaagaatgagaaa-acaggtaa----aaataaaaatactgtctttgtgacatgg---tta

RP9P gaagaatggagaaaacaggtaaaaataaaaata**agtactgtctttgtgatgtggt**tattc

RP9 ttcatcatcctatactgcttttgaatgacatatttatctaagcaactgttgtaagttaaa

RP9P atgatccttccatattgcttttgaatgacatatttatctaagcaactgttgtaagttaaa

RP9 gctaaaactcactgttctttgatcaattttgaatgtttagGATACAGCAGTTAAAACAGT

RP9P gctaaaactgactgttctttgatcaattttgaatgtttagGATACAGCAGTTAAAACAGT

RP9 TACTGGAGGATTCTACCTCAGATGAAGATAGGAGCAGCTCCAGTTCCTCTGAAGGTAAAG

RP9P TACTGGAGGATTCTACCTCAGGTGAAGATAGGAGCAGCTCCAGTTCCTCTGAAGGTAAAG

RP9 AGAAACACAAGAAAAAGAAGAAGAAAGAAAAGCATAAGAAAAGGAAGAAAGAAAAGAAAA

RP9P AGAAACACAAGAAAAAGAAGAAGAAAGAAAAGCATAAGAAAAGGAAGAAAGAAAAGAAAA

RP9 AGAAGAAAAAACGGAAGCACAAATCTTCCAAGTCAAATGAGGGTTCTGACTCAGAGTGAC

RP9P AGAAGAAAAAACGGAAGCACAAATCTTCCAAGTCAAATGAGGGTTCTGACTCAGAGTGAC

RP9 AAGGATGTGACTTGTTCAACATTCTCTTCTCAAACACTGACCAAGGAACAGAGGAAGATG

RP9P AAGGATGTGACTTGTTCAACATTCTCTTCTCAAACACTGACCAAGGAACAGAGGAAGATG

RP9 CAGTCAGAGAAAGCAGCAGGATAGAGACGCCGAGAGAGGAGTATATGTGGGTCACAGCAG

RP9P CAGTCAGAGAAAGTAGCAGGATAGAGACGCCGAGAGAGGAGTATATGTGGGTCACAGCAG

RP9 TGAGCTCCCACCCGCCTTGCAGTGAAGATGTGACCCCAGGAGAGGGAGTGTCTCCTTCCA

RP9P TGAGCTCCCACCCGCCTTGCAGTGAAGATGTGACCCCAGGAGAGGGAGTGTCTCCTTCCA

RP9 GGTGCTAGCTCTGGACAGCAGCTGATTTTAGGCAGGAAAGTTTCTTCATCGTTGTCCTCC

RP9P GGTGCTAGCTCTGGACAGCAGCTGATTTTAGGCAGGAAAGTTTCTTCATCGTTGTCCTCC

RP9 CTGCTGGTCACATGAGTTTACGATTCCTTTGAAGTGTCTCCCACAGGGTGGCAGGACTGG

RP9P CTGCTGGTCACATGAGTTTACGATTCCTTAGAAGTGTCTCCCACAGGGTGGCAGGACTGG

RP9 GAGAATCTCTGAGGCGTGTCTTCCAGGCCCTCCCACAGCTTGTGCCCTCCACAGTGTGGA

RP9P GAGAATCTCTGAGGCGTGTCTTCCAGGCCCTCCCACAGCTTGTGCCCTCCACAGTGTAGA

RP9 CTCAGG**TCCCATAGACATCAGGCTGG**AGTCTTCTCTGTTGTT------------------

RP9P CTTGAACACTTGGCCTCATGTGATCCTTCCACCTTGGCCTCCCAAA**GCATTGGGATTACA**

RP9 ------------------------------------------------------------

RP9P **GGCGAG**AGCCACTGGTTGGCTAAAATATTTATATTTACCTGACATGATTTGTTGATTTGG

RP9 ------------------------------------------------

RP9P GGGCTAGGGAGTTCTTAAAACAACAATAAAAAATCTTCTGTGGCTGCA

Primers to amplify RP9 Ex6 and not RP9P

agacatgattagagagaggtgga, RP9_Ex6F

CCAGCCTGATGTCTATGGGA, RP9_Ex6R

Size = 1040bp

**Primers to amplify RP9P and not RP9**

Agtactgtctttgtgatgtggt, RP9P-F

CTCGCCTGTAATCCCAATGC, RP9P-R

Size = 840bp

**Supplementary figure 3.** DNA sequence comparative alignment between *RP9* and *RP9P* for a region centered upon the *RP9* c.509A>G; p.(D170G) variant, which is highlighted in yellow. Non-matching nucleotides are shaded. Note the non-reference A for *RP9* at c.509A>G; p.(D170G) corresponds with the reference G in the homologous *RP9P* sequence.

| Sample | %bases >=Q30 | Mean cov. | Yield (Mb) | %bases >=10x | %bases >=20x | Coverage over c.410A>T, p.H137L |
| --- | --- | --- | --- | --- | --- | --- |
| 289 | 75.79 | 28.65 | 105010 | 95.01 | 77.35 | A:12, T:6 (18) |
| 296 | 82.48 | 35.92 | 138461 | 96.18 | 89.20 | A:13, T:16 (29) |

**Supplementary Table 1** – Sequencing and alignment metrics from whole genome sequencing of the RP9 family members.

| **Position (GRCh38)** | **REF** | **ALT** | **Gene** | **Whole Genome Sequencing** | | | **100kGP cases** | | **Additional cases** | |
| --- | --- | --- | --- | --- | --- | --- | --- | --- | --- | --- |
|  |  |  |  | **Control** | **RP9-289** | **RP9-296** | **Case 1** | **Case 2** | **RP9-1618** | **RP9-5477** |
| chr7:32696283 | A | G | *DPY19L1P1* | A/A | A/G | A/G | A/G | A/G | A/G* | A/G* |
| **chr7:33096550** | **T** | **A** | ***RP9*** | **T/T** | **T/A** | **T/A** | **T/A** | **T/A** | **T/A** | **T/A** |
| chr7:33769044 | T | A |  | T/T | T/A | T/A | T/A | T/A | . | . |
| chr7:33969722 | A | G | *BMPER* | A/A | A/G | A/G | A/G | A/G | . | . |
| chr7:34559955 | A | C | *NPSR1-AS1* | A/A | A/C | A/C | A/C | A/C | . | . |
| chr7:34802348 | G | C | *NPSR1* | G/G | G/C | G/C | G/C | G/C | . | . |
| chr7:34901635 | A | G |  | A/A | A/G | A/G | A/G | A/G | . | . |
| chr7:34968054 | A | C | *DPY19L1* | A/A | A/C | A/C | A/C | A/C | . | . |
| chr7:35062468 | G | A |  | G/G | G/A | G/A | G/A | G/A | . | . |
| chr7:35120873 | C | A | *DPY19L2P1* | C/C | C/A | C/A | C/A | C/A | . | . |
| chr7:35162505 | C | A | *DPY19L2P1* | C/C | C/A | C/A | C/A | C/A | C/A* | C/A* |

**Supplementary Table 2.** Genotypes for the same rare genomic variants detailed in table 1, in two members of the original linked family and two cases identified in genome sequence from GEL, with limited data also available from the two additional cases identified through exome sequencing. All six cases of RP carrying RP9 p.H137L share the same haplotype of 10 rare variants, each found at less than 0.1% of the population. Flanking variants in the additional cases (highlighted by an *) have been validated using sanger sequencing (supplementary figure 1).

| **Phenotypes in cases of RP9 p.D170G in 100KGP** | | | | | | | |
| --- | --- | --- | --- | --- | --- | --- | --- |
| **Genome version** | **Genomic location** | **SNP** | **Gene** | **Individual (Sex)** | **Solved?** | **Disorders** | **Affection** |
| GRCh38 | 7:33095391 | rs104894039 | RP9 | 1 (Female) | Yes |  | Unaffected |
|  |  |  |  | 2 (Female) | No | Respiratory disorders (Familial pulmonary fibrosis) | Affected |
|  |  |  |  | 3 (Female) | No | Endocrine disorders (IUGR and IGF abnormalities) | Affected |
|  |  |  |  | 4 (Male) | No |  | Unaffected |
|  |  |  |  | 5 (Male) | No |  | Unaffected |
|  |  |  |  | 6 (Female) | No | Tumour syndromes (Familial breast cancer) | Affected |
|  |  |  |  | 7 (Female) | No |  | Unaffected |
|  |  |  |  | 8 (Female) | No | Ultra-rare disorders ( Undescribed monogenic disorders) | Affected |
|  |  |  |  | 9 (Female) | No |  | Unaffected |
|  |  |  |  | 10 (Female) | No | Dermatological disorders (Erythropoietic protoporphyria, mild variant) | Affected |
|  |  |  |  | 11 (Female) | Yes |  | Unaffected |
|  |  |  |  | 12 (Male) | No | Tumour syndromes (Genodermatoses with malignancies) | Affected |
|  |  |  |  | 13 (Female) | No | Tumour syndromes (Genodermatoses with malignancies) | Affected |
|  |  |  |  | 14 (Female) | No | Tumour syndromes (Genodermatoses with malignancies) | Affected |
|  |  |  |  | 15 (Female) | Yes | Haematological and immunological disorders (Primary immunodeficiency) | Affected |
|  |  |  |  | 16 (Female) | No |  | Unaffected |
|  |  |  |  | 17 (Female) | No |  | Unaffected |
|  |  |  |  | 18 (Female) | No |  | Unaffected |
|  |  |  |  | 19 (Female) | No |  | Unaffected |
|  |  |  |  | 20 (Female) | Unknown |  | Unaffected |
|  |  |  |  | 21 (Male) | Unknown | Hearing and ear disorders (Congenital hearing impairment) | Affected |
|  |  |  |  | 22 (Female) | No |  | Unaffected |
|  |  |  |  | 23 (Male) | No | Ophthalmological disorders (Cataracts) | Affected |
|  |  |  |  | 24 (Female) | No |  | Unaffected |
|  |  |  |  |  |  |  |  |
| GRCh37 | 7:33135003 | rs104894039 | RP9 | 25 (Female) | No | Neurology and neurodevelopmental disorders (Intellectual disability) | Affected |
|  |  |  |  | 26 (Male) | No |  | Unaffected |

**Supplementary Table 3.** Cases of RP9 p.D170G in 100KGP and their associated phenotypes.
